# Computational-Based Study of QuEChERS Extraction of Cyclohexanedione Herbicide Residues in Soil by Chemometric Modeling

**DOI:** 10.3390/molecules23082009

**Published:** 2018-08-11

**Authors:** Juan José Villaverde, Beatriz Sevilla-Morán, Carmen López-Goti, José Luis Alonso-Prados, Pilar Sandín-España

**Affiliations:** Unit Plant Protection Products, DTEVPF, INIA. Crta. La Coruña, Km.7.5, 28040 Madrid, Spain; juanjose.villaverde@inia.es (J.J.V.); bsmoran@inia.es (B.S.-M.); lgoti@inia.es (C.L.-G.); prados@inia.es (J.L.A.-P.)

**Keywords:** pesticides, cyclohexanedione oxime herbicides, soil matrix, QuEChERS, chemometric modeling, factorial design, matrix effect

## Abstract

Assessment of two buffered QuEChERS (quick, easy, cheap, effective, rugged, and safe) versions (i.e., citrate and acetate) modified by including methanol to recover the residues of three cyclohexanedione oxime (CHD) herbicides and three of their byproducts from agricultural soil was performed. In this context, a full second-order face-centered factorial experimental design was developed to quantify the influences of the main five variables (i.e., extraction time, water content, soil weight, and extraction solvent volume and composition) on the target compound recoveries. The fitting equations satisfactorily described the extraction process behavior. The mathematical models also showed the most influencing independent variables (i.e., extraction solvent composition and soil weight). Handling simpler expressions was possible with the acetate QuEChERS but not with the citrate QuEChERS. The recoveries of the CHD residues were close to 100% after performing the extraction under suitable conditions. Furthermore, dispersive solid-phase extraction (dSPE) clean-up steps were assessed to reduce the matrix effect in mass spectrometry. In this sense, the citrate QuEChERS in combination with the PSA + C18 clean-up step was the best option for the extraction of CHD residues.

## 1. Introduction

Pesticides are ubiquitous contaminants in the environment [1,2,3], as field soils are a frequent receptor of these compounds when they are applied to crops [4,5]. Not only pesticides but also their degradation products—which result from the environmental exposure of pesticides to microorganisms, light, etc.—are found in soil. Therefore, the establishment of reliable methods for analyzing pesticide residues in soils is of utmost importance to assess their effects on this environmental compartment [3,6,7]. However, the determination of pesticide residues in soils is not a simple task due to the complexity and heterogeneity of this matrix as well as to the low concentrations of pesticide residues commonly found.

Cyclohexanedione oxime (CHD) herbicides are thermolabile and low volatility pesticides used for postemergence control of annual and perennial grass weeds in broad-leaved crops such as sugar beet, soybean, or oilseed rape. In general, CHD herbicides are rapidly degraded under different environmental conditions, including biotic and abiotic processes, leading to the formation of new byproducts [8,9,10]. These new compounds may pose a potential threat to succeeding crops or nontarget organisms. Indeed, different studies have suggested the possibility that some of the byproducts together with the parent CHD herbicide induce phytotoxic effects on grasses [11] and harmful effects on animals and the environment [8,10]. As these herbicides are applied at early growth stages of plants, a large fraction of them directly reach soil, where they are subjected to degradation processes and their residues may remain for a while. However, to date, few papers have reported the simultaneous determination of CHD herbicides and their byproducts in environmental matrices [12,13,14,15,16].

Recently, the QuEChERS (quick, easy, cheap, effective, rugged, and safe) method has become one of the most widely used techniques for sample preparation in pesticide analysis. This method helps overcome some obstacles derived from the coextraction of matrix components, such as coelution, chemical background noise or signal enhancement/suppression. Although this methodology was initially developed for extracting and cleaning up pesticides from plant-based commodities (i.e., fruits or vegetables) [1,17,18], QuEChERS use has been extended to other matrices, including environmental matrices such as soil [12,19,20]. However, the application of QuEChERS to soil matrices involves the optimization of different parameters. These optimizations are often performed by sequential analysis of individual variables, which is time consuming and increases the analysis costs [12,16,19]. In this context, chemometrics could help overcome these drawbacks, thereby reducing the number of experiments and maximizing the information obtained [21].

To our knowledge, chemometric optimization of CHD residue extraction from soil has not been performed before [22]. In this sense, models to ensure the efficient extraction of CHD residues from soil and to fix operational conditions leading to optimal results would be established for the first time. Using a statistical approach systematically to interpret the role played by QuEChERS parameters and the physicochemical properties and chemical structures of analytes on the extent of recovery, is a further required step toward achieving a better use of QuEChERS technique in pesticide residue analysis. Moreover, implementation of a methodology like this, as requirement for the analytical methods with QuEChERS and developed for pesticide risk assessment, could provide further information to the reference laboratories of pesticide residues for a better adoption of a multiresidue method in their facilities. Currently, these laboratories, such as the European Reference Laboratories, have a growing demand for high sample throughput. In this sense, models with the capacity for describing the recovery of pesticide residues in function of key variables would also be of special significance to minimize, for example, the analysis time, quantity of sample (e.g., soil) and solvent expense without compromising the analytic performance, especially when the facility to recover the different pesticides is not the same.

**Figure 1:**
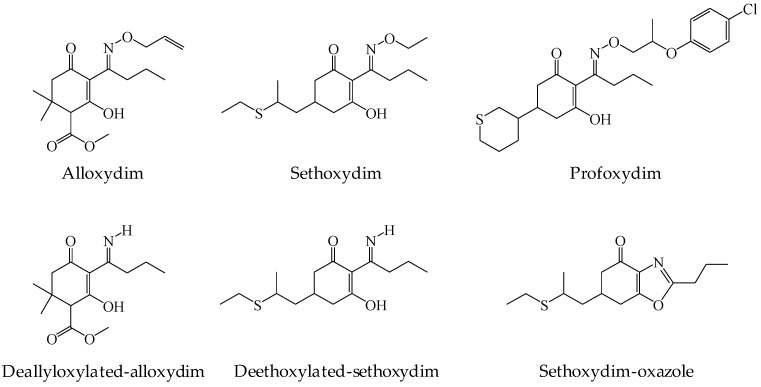
Chemical structure of the selected CHD herbicides (i.e., alloxydim, sethoxydim and profoxydim) and three of their degradation products (i.e., deallyloxylated-alloxydim, deethoxylated-sethoxydim and sethoxydim-oxazole).

With this background, a systematic multivariable analysis was performed to optimize two versions of the QuEChERS method (i.e., citrate and acetate) modified by including methanol for the extraction of three CHD herbicides (i.e., alloxydim, sethoxydim and profoxydim) and three of their degradation products (i.e., deallyloxylated-alloxydim, deethoxylated-sethoxydim and sethoxydim-oxazole) from soil (Figure 1). The studied independent variables were extraction time (ET), water content (WC), soil weight (SW), solvent extraction volume (EV) and the solvent mixture of acetonitrile/methanol by controlling the proportion of acetonitrile (AM).

## 2. Results and Discussion

### 2.1. Optimization of the Chromatographic Method

First, the chromatographic conditions were optimized to achieve the optimum separation of the six target compounds.

Due to the acidic character of the target compounds, the addition of formic acid helps improve the protonation of the target compounds and hence the peak shape. Thus, the best results were obtained using a mixture of acetonitrile-water in which 0.1% formic acid was used to acidify the mobile phase. The addition of a higher concentration of formic acid did not improve the separation or mass ionization.

Regarding the stationary phase, three C18 columns with different particle types, lengths and particle sizes were compared: Nova-Pak^®^ C18 (4 µm, 3.9 × 150 mm), Atlantis T3 (3 μm, 4.6 × 150 mm) and Kinetex^®^ 2.6 µm C18 100 Å (100 × 4.6 mm). For the three columns tested, adequate separations of the six target compounds (resolution >1.5) in relatively short run times (7–11 min) were obtained. The Kinetex column was selected for the chromatographic analysis since it shows excellent resolution at the beginning of the chromatographic separation, where the degradation products eluted (i.e., deallyloxylated-alloxydim, deethoxylated-sethoxydim and sethoxydim-oxazole) (Table S3).

Regarding the mass analyzer, the best mass response for all target compounds was observed in Electrospray Ionization (ESI) positive ion mode. Moreover, the most intense ions for the target compounds corresponded to their protonated molecule ions [M + H]^+^, so these ions were selected for quantification purposes/matrix study (Table S4).

### 2.2. Modeling of the QuEChERS Extraction Method

For the development of the factorial design, photodiode array detector (DAD) was selected as the detector due to the variability of the matrix effect on the mass response observed under the different extraction conditions. Thus, the use of mass spectrometry (MS) detectors would make it difficult to model the QuEChERS method since it would involve the preparation of 90 different external matrix-matched calibrations.

The aim of the factorial design is to identify the key variables and possible synergistic effects controlling the process [21,23], which allow modeling of the QuEChERS extraction to maximize the recoveries of CHD residues from soil. Bruzzoniti et al. [24] have already highlighted the necessity of using chemometric tools to contribute to a better understanding of the extraction process in soil.

**Figure 2:**
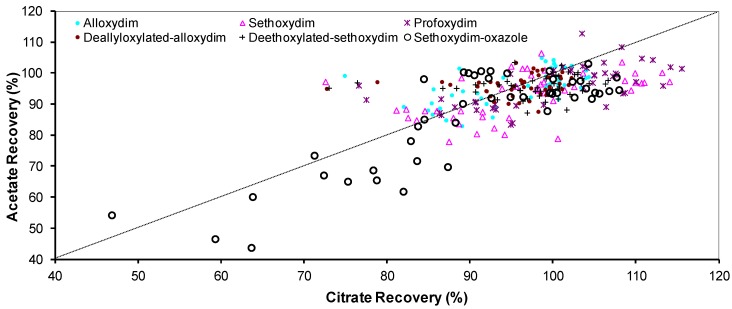
Experimental results and correlation between citrate and acetate quick, easy, cheap, effective, rugged, and safe (QuEChERS) recoveries for each of the extraction conditions assessed.

Tables S1 and S2 show the recoveries obtained for the CHD residues during the factorial design of the QuEChERS extraction. The recoveries for the majority of compounds are usually within the acceptable range of 70–110% at the EU level for analytical methods according to Regulation (EU) No 283/2013 (Figure 2, Tables S1 and S2). An exception to this generality is observed in several citrate tests for sethoxydim and profoxydim for which the recoveries surpass the regulated threshold. These observations seem to be in agreement with those made by Lehotay et al. [25], who found that the acetate-buffered version of QuEChERS gives better recoveries for most of the pesticides studied.

Figure 2 shows a large dispersion regarding the recoveries of the target compounds. Therefore, precise selection of the extraction conditions is required to obtain suitable recoveries.

Equation 1 fitted the experimental data well by means of least squares multiple regressions, and the resultant polynomial models are shown in Table 1 and Table 2 together with the significance levels of the factors and the statistics parameter displaying the goodness of fit.

#### 2.2.1. Factorial Design of Citrate QuEChERS

**Figure 3:**
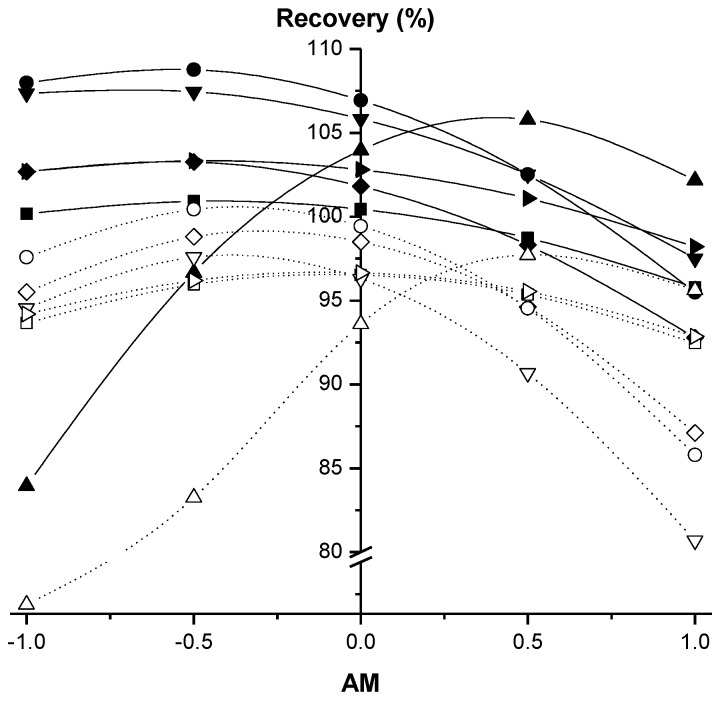
Cross sections of the response surfaces for recoveries fitted to the experimental data. Behavior of the acetonitrile/methanol (AM) variable is shown for each of the six compounds studied when all other variables have normalized values of zero. Bold and empty symbols show the results for the citrate and acetate QuEChERS, respectively. Symbols show the results for alloxydim (◆◇), sethoxydim (▼▽), profoxydim (●○), deallyloxylated-alloxydim (■□), deethoxylated-sethoxydim (▶▷) and sethoxydim-oxazole (▲△).

**Figure 4:**
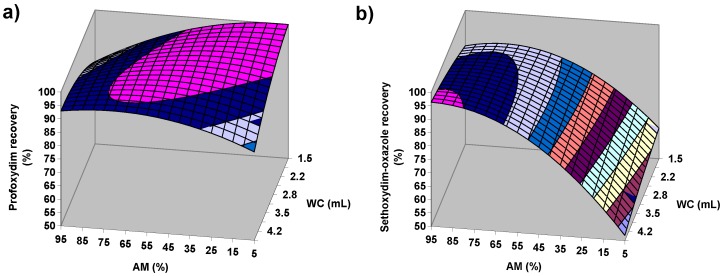
Effects of acetonitrile/methanol (AM) and water content (WC) on the recovery of profoxydim (**a**) and sethoxydim-oxazole (**b**) by QuEChERS citrate (SW = 5.8 g, EV = 8 mL, ET = 1 min).

In the case of citrate QuEChERS, AM is the variable that exerts the largest influence on recoveries at a confidence level of 95% (Table 1). For all target compounds except for sethoxydim-oxazole, when AM increases, the recoveries decrease (Table 1), especially in the high AM range (Figure 3). However, a high value of WC can change this trend for CHD herbicides, as shown in Figure 4a, where the typical shape of a response surface for the target compounds is depicted. The negative sign of the fitting parameter b_WC_ for these CHD herbicides (Table 1) is responsible for the response surface falling in the area of high WC. This fact seems opposite to the assumed importance of the water to the successful extraction of the pesticides from the soil [19,26,27,28]. However, b_WC_ is significant only in the best case at a confidence level of 80% (Table 1), and these observations may be related to the inherent error associated with the experimental procedure. In this sense, the decrease in recoveries can be easily offset by increasing the AM slightly (Figure 4a). In the case of sethoxydim-oxazole, a significant opposite trend is observed for AM in whole range of WC with a comparatively higher intensity (Table 1, Figure 3 and Figure 4). The comparison performed in Figure 4 between sethoxydim-oxazole and, for example, profoxydim, clearly shows the highlighted differences between sethoxydim-oxazole and the rest of the compounds.

Moreover, an important significant value at a 95% confidence level for sethoxydim-oxazole is also observed in the fitting parameter associated with the quadratic term of AM (b_AM-AM_) with a negative value (Table 1), which causes the existence of maxima in the graphical representation of the AM and recovery values (Figure 3 and Figure 4b). Therefore, good recoveries of sethoxydim-oxazole are achieved at high AM values, and its recoveries are very sensitive to small variations of the AM variable.

On the other hand, two important interactions between AM and WC and between AM and EV (b_AM-WC_ and b_AM-EV_, respectively) are observed on the target compound recoveries (Table 1). Thus, except for sethoxydim-oxazole, an important positive effect is observed for the fitting parameter b_AM-WC_ (Table 1), which could be considered a synergistic effect on the recoveries of all target compounds due to the negative effect and high importance of the individual fitting parameter b_AM_ and the low importance of the individual fitting parameter b_WC_ in the polynomial model (Table 1). This effect of the b_AM-WC_ fitting parameter on favoring the dissolution of the polar CHD residues might come from an increase in polarity and protic characteristics upon reaching a sufficient threshold in the extraction medium when acetonitrile, in combination with methanol, is added and combined with water [29,30]. Conversely, a negative effect on the target compound recoveries is observed for the fitting parameter b_AM-EV_ (Table 1). However, in this case, the AM effect on the target compound recoveries is partially compensated for by the EV (i.e., acetonitrile and methanol) by adding more quantity of methanol, that is, a solvent that is polar and protic but less so than water [29,30], thereby diminishing the negative significance of the fitting parameter b_AM-EV_ regarding the significance of the individual fitting parameter b_AM_. The fitting parameter b_AM-EV_ seems to indicate a synergistic effect in the case of sethoxydim-oxazole: both independent variables, i.e., AM and EV are significant at a 0.05 level with a positive effect on sethoxydim-oxazole recovery, while their interaction is also significant at a 0.05 level but with a negative effect in this case (Table 1). This effect might be observed when the medium becomes sufficiently polar and protic. The interaction of the three variables, i.e., AM, EV and WC (b_AM-EV-WC_) (Table 1), with negative effects on the recoveries of the CHD residues should mainly result from acetonitrile.

#### 2.2.2. Factorial Design of Acetate QuEChERS

In this case, a significant global influence is given by SW at a confident level of 95% on the recoveries for all target compounds (Table 2). The greater influence and tendency observed with the variable AM in the citrate QuEChERS appears to be maintained in the acetate version for all compounds except for the dealkoxylated byproducts (i.e., from alloxydim and sethoxydim). Regarding the WC, this variable has more influence on the degradation products of sethoxydim (i.e., deethoxylated- and oxazole- sethoxydim) at a confidence level of 95%, followed by profoxydim and deallyloxylated-alloxydim at a confidence level of 90%. Moreover, the WC shows a higher positive influence on recoveries of all target compounds in the acetate QuEChERS versus the citrate QuEChERS.

**Figure 5:**
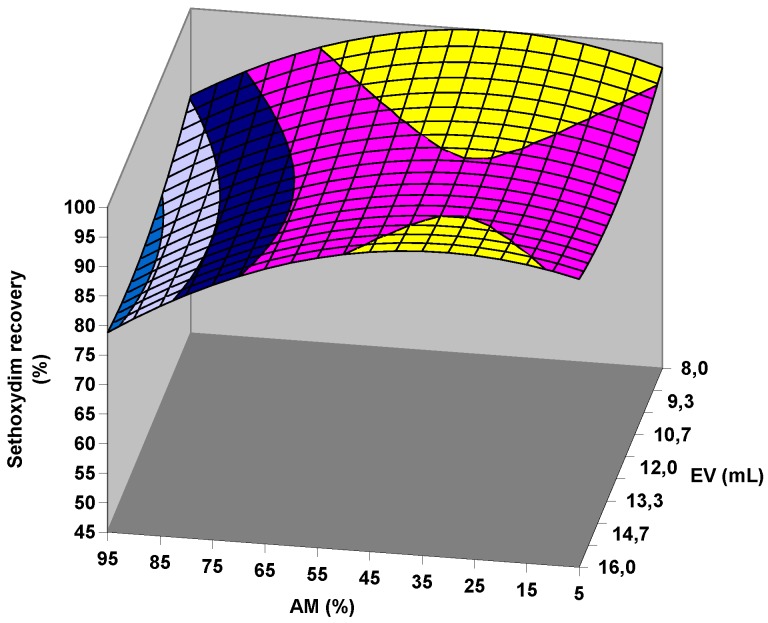
Effects of acetonitrile (AM) and extraction solvent volume (EV) on the recovery of sethoxydim by QuEChERS acetate (SW = 5.8 g, WC = 1.5 mL, ET = 3 min).

Acetate QuEChERS, similar to the citrate version, shows maxima in the graphical representations of AM versus recovery values (Figure 3). However, in this case, significance is observed in the AM quadratic term (b_AM-AM_) for all target compounds (Table 2). The EV quadratic terms (b_EV-EV_) for alloxydim and sethoxydim are also statistically significant (0.05 and 0.10 level, respectively) but in this case with a positive sign (Table 2). Therefore, both AM and EV should be taken into special consideration during the extraction of sethoxydim and alloxydim because maxima and minima are observed when both variables are depicted together, resulting in maximal recoveries at both extremes of the EV tested and with medium or low AM (see an example in Figure 5). This effect is not so evident for the rest of the target compounds. In contrast to acetate, citrate QuEChERS did not exhibit this trend since all quadratic terms were negative (Table 1); i.e., there were no minima. In this sense, Figure 5 shows that once, for example, the maximum extraction at the lowest EV is reached, if the AM is kept constant while the EV is increased, then the greater polar and protic effect from the water of the medium relative to the lower polar and protic effect that a higher EV implies may “vanish” since methanol and acetonitrile are less polar and protic than water. However, the amount of methanol in the medium is eventually sufficient to compensate for the lower polar and protic power of the extraction medium; thus, the maximum extractions are reached again. This effect, the importance of the individual fitting parameter b_WC_ (Table 2) and the synergistic effect favoring the target compound recoveries at a 0.05 level between SW and WC, which is observed when the individual fitting parameters b_SW_ and b_WC_ are compared with the fitting parameter b_SW-WC_ (Table 2), clearly denote the importance of water in the case of the acetate QuEChERS. A plausible explanation for the synergistic effect among SW and WC might come from soil rehydration, which improves the access to the soil pores and, consequently, the pesticide residue partitioning process toward acetonitrile/methanol. Competition between water and some pesticides for adsorption sites of soil humic substances by forming hydrogen bonds can also be a simultaneous process that promotes pesticide desorption. These two hypotheses were also provided by other authors [27,31] after observing an improvement in the recoveries of pesticides with different QuEChERS procedures after soil hydration.

A high ET when the AM is increased has shown detrimental effects on the recoveries of the target compounds (see fitting parameter b_AM-ET_ in Table 2), possibly as a consequence of increased diffusion and adsorption of the CHD residues in/on solids, i.e., soil and salts of acetate QuEChERS.

Finally, the different behavior of sethoxydim-oxazole in both extraction QuEChERS (i.e., citrate and acetate) seems to indicate that the absence of a keto-enol equilibrium in sethoxydim-oxazole, which conversely occurs in the rest of target compounds (see chemical structures in Table S4), influences its interaction either with the soil or with the solvents.

### 2.3. Handling of the Polynomial Models

To handle simpler expressions, the nonsignificant terms (e.g., below the 95% confidence level) of the obtained empirical mathematical equations could be removed [32]. However, a proper demonstration of the suitability of this action is required. In this sense, to avoid unreal distortions as a consequence of an improper definition of the b_0_ constant, a study to assess the effect of removing terms according to their significance level from the empirical correlations was planned. Table 3 shows the existing deviations between the experimental recoveries and the polynomial model results determined according to the minimum fixed confidence level established (i.e., confidence level ≥95%, ≥90%, ≥80%, ≥70% and ≥0% (full equation)).

The deviations obtained after removing all terms of the polynomial models below the 95% confidence level are always below 10% for the acetate QuEChERS. However, this action is not suitable in the case of citrate QuEChERS. In fact, at the 70% confidence level, none of the six target compounds are determined with acceptable deviations within any of the ranges studied for the different independent variables (Table 3). Moreover, the deviations between the experimental recoveries and the polynomial model results were lower for the acetate QuEChERS than for the citrate QuEChERS before and especially after removing the nonsignificant terms. The lower citrate response surface curvature, defined by less significant quadratic and interaction terms (Table 1 and Table 2), explains the differences obtained with both QuEChERS. These results clearly indicate that a case-by-case study should be performed if the intention is to remove terms from the polynomial models. However, keeping all terms in the equations does not make the model incorrect; instead, it makes the model more complex but also more accurate.

On the other hand, if the R values showing the goodness of fit are compared, it is clear that the R values are not in agreement with the range of deviations. In this sense, a low R in the QuEChERS acetate does not mean a great deviation, e.g., deallyloxylated-alloxydim and deethoxylated-sethoxydim at the 95% confidence level. This is due to a low dispersion in the recovery values, with response surfaces that cross the whole cloud of recovery points in a suitable way, achieving low deviations relative to the experimental results. An opposite situation is observed, for example, with sethoxydim-oxazole in the citrate QuEChERS at any confidence level (Table 3). In this case, the wide range in the recovery values provides a high R, while the presence of some individual recoveries far away from the response surfaces is responsible for the worst deviations [33]. Therefore, the regression parameter R alone should not be considered sufficient to establish the accuracy of the models.

### 2.4. Confirmation of Extraction Conditions and Clean-Up Assessment

According to the experimental results previously discussed, the polynomial models obtained for citrate and acetate QuEChERS (Table 1 and Table 2) provide different extraction conditions to achieve recoveries of 100% for the target compounds when full equations are used. Therefore, the best experimental management conditions were selected for the extraction. In this sense, as an additional criterion, the minimum values of the variables were selected for the confirmation studies. Table 4 shows the final optimized extraction conditions determined by the polynomial models and the experimental recoveries obtained.

Despite advances in sample preparation methods, pesticide residues are often found at levels too low to be detected by DAD detectors in environmental matrices. In this regard, mass spectrometers are known to provide a higher sensitivity and selectivity, but the matrix effect is a major drawback for the quantitative determination of analytes by this technique [3,34]. Thus, once the best conditions for citrate and acetate QuEChERS extraction were selected (Table 4), the matrix effect was evaluated under these conditions to select the best combination of QuEChERS extraction/clean-up step (PSA and PSA-C18) to analyze CHD residues by HPLC-MS.

**Figure 6:**
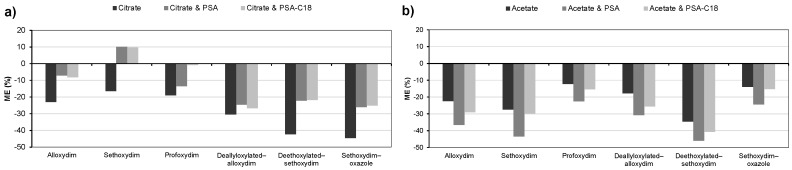
Matrix effects (ME) observed on the target compounds using citrate (**a**) and acetate (**b**) QuEChERS extraction kits alone and in combination with PSA or PSA-C18 clean-up steps for soil pretreatment and HPLC-MS for the determination.

As illustrated in Figure 6, suppressive effects on MS signals are observed for all the target compounds when either the QuEChERS citrate or acetate extraction kit is used without a clean-up step. This signal suppression is higher with citrate QuEChERS than with the acetate version. Although the best results were observed with the acetate QuEChERS, the coelution of soil components makes it necessary to subtract the matrix blank signal for quantification and to thoroughly clean the chromatographic column after each injection.

To reduce the matrix effect, two different clean-up QuEChERS kits were used, PSA and PSA-C18. PSA removes sugars and fatty acids, whereas the C18 sorbent effectively traps and removes lipids, starch, and humic substances [19,35,36]. In this study, two opposite results were observed: whereas citrate QuEChERS extraction combined with PSA or PSA-C18 clean-up led to a reduction in the suppressive matrix effect on all the target compounds (Figure 6), the combination of acetate with both clean-up QuEChERS led to a higher suppression of the MS signals. Notably, with the citrate QuEChERS, a similar reduction in the matrix effect was observed for both clean-up PSA and PSA-C18. Regarding the acetate QuEChERS, the coelution of soil components was reduced when PSA-C18 was used as clean-up step.

According to the findings mentioned above, the best combination for the determination of CHD residues from soil entails extraction with citrate QuEChERS followed by the PSA-C18 clean-up step. However, since the matrix effect is maintained above 20%, the use of external matrix-matched standards is recommended [37,38].

## 3. Materials and Methods

### 3.1. Chemicals

Analytical standards of the herbicides alloxydim-sodium (98.0% purity) and profoxydim-lithium (99.2% purity) were purchased from Pestanal^®^ (Steinheim, Germany), whereas sethoxydim-lithium (99.3% purity) was supplied by BASF Ltd. (Limburgerhof, Germany). The degradation products sethoxydim-oxazole (95.5% purity) and deethoxylated-sethoxydim (97.0% purity) were purchased from ChemService Inc. (West Chester, PA, USA). The byproduct deallyloxylated-alloxydim was obtained in our laboratory, and the methodology was explained elsewhere [39]. Briefly, aqueous solutions of the herbicide alloxydim were irradiated for 110 h with a xenon arc lamp (λ > 290 nm; 750 W m^−2^), and the degradation product was concentrated by solid-phase extraction (ENV+ cartridge, elution with 3 × 2 mL methanol) (Westminster, UK).

For sample extraction, two different commercial QuEChERS were used: QuEChERS-Citrate (4.0 g magnesium sulfate, 1.0 g sodium chloride, 0.5 g sodium citrate dibasic sesquihydrate, 1.0 g sodium citrate tribasic dihydrate) and QuEChERS-Acetate (6 g magnesium sulfate, 1.5 g NaOAc). For the clean-up step, two different commercial QuEChERS were used: QuEChERS-PSA (900 mg magnesium sulfate, 150 mg PSA) and QuEChERS-PSA-C18 (900 mg magnesium sulfate, 150 mg PSA, 150 mg C18). All QuEChERS were purchased from HPC Standards GmbH (Cunnersdorf, Germany).

Acetonitrile (HPLC superGRAD grade) and methanol (HPLC grade) were acquired from Macron Fine Chemicals (Gliwice, Polland). The water used for the LC mobile phase and the aqueous solutions were purified with a Millipore system (Milli-Q-50 18 mΩ) (Molsheim, France).

Stock solution containing a mixture of the six target compounds (i.e., alloxydim, deallyloxylated-alloxydim, sethoxydim, sethoxydim-oxazole, deethoxylated-sethoxydim and profoxydim) was prepared in methanol at a concentration of 500 mg L^−1^. This solution remained stable for ten days when stored in the dark at −18 °C. Working solutions were prepared daily by dissolving the appropriate amount of the composite stock solution in acetonitrile/methanol or in matrix extracts.

### 3.2. Soil Samples

The soil used in the experiments was collected from an agricultural field located in Sevilla province (southwest of Spain) with no history of CHD herbicide application. The soil was taken from the top layer (0–20 cm), and after being air-dried at room temperature, it was passed through a 2 mm sieve and stored at 4 °C until use. Soil texture was determined by sedimentation using the pipette method [40]. The organic carbon an nitrogen content were determined according to the Walkley and Black method [41] and the Kjeldahl method [42], respectively. Soil pH was measured in a 1:2.5 (*w*/*v*) soil/deionized water mixture. Soil at the site was classified as sandy clay loam containing approximately 32% sand, 55% silt, and 13% clay, with organic carbon = 9.3 g kg^−1^ dw, N_Kjeldahl_ = 0.89 g kg^−1^ dw and pH = 7.2.

The soil samples were spiked with the stock solution containing 10 mg kg^−1^ dw of each of the six target compounds, which corresponds to a field application rate of 200 mg ha^−1^ of each target compound, close to the recommended application doses for CHD herbicides. The mixture was vigorously shaken for 1 min with an Intelli-Mixer device (JP SELECTA S.A., Barcelona, Spain) and subsequently kept in the dark for 2 h to allow the interaction between the target compounds and the matrix.

### 3.3. QuEChERS Procedure

The general QuEChERS method was as follows: a portion of soil was weighed (SW) into a 50 mL centrifuge tube, and water was added (WC). Afterwards, different EVs of solvent mixtures (acetonitrile/methanol (AM)) were added, and the mixture was shaken for 1 min with the Intelli-Mixer device. The content of the commercial extraction QuEChERS (citrate or acetate) was added, and the mixture was immediately shaken for different ETs and subsequently centrifuged for 5 min at 4000 rpm and 4 °C.

For the clean-up step by dispersive solid-phase extraction (dSPE), an aliquot of the supernatant of the QuEChERS extraction tube (2 mL) was transferred into a 15 mL centrifuge tube containing commercial clean-up QuEChERS (PSA or PSA-C18). The tube was shaken for 1 min and centrifuged for 5 min at 4000 rpm and 4 °C, and the resulting supernatant was filtered using a 0.45 µm PVDF syringe filter for chromatographic injection.

### 3.4. Factorial Design of QuEChERS Extraction

The main independent variables selected during the QuEChERS extraction and their working ranges were the following: ET, (1–3 min.); WC, (1.5–4.5 mL); AM, (5–95%); EV, (8–16 mL) and SW, (2.4–5.8 g). The SW range was maintained below 6 g to facilitate the management of a representative soil sample in the lab, and the minimum EV was selected as a function of the wettability of the QuEChERS kit and the concentrations of the target compounds in the final extracts.

3^5^ full second-order face-centered factorial experimental designs with three center points were performed to determine the influence of selected factors and their effects on the extraction of target compounds from soil [33].

The general model for recovery is the following polynomial equation:

(1) $$Y=b_{0}+\sum_{i=1}^{5}b_{i}X_{i}+\sum_{i=1}^{5}b_{{}^{\mathrm{ii}}}X_{i}^{2}+\sum_{i<j}^{5}b_{\mathrm{ij}}X_{i}X_{j}+\sum_{i<j<k}^{5}b_{\mathrm{ijk}}X_{i}X_{j}X_{k}$$

where Y represents the dependent variable or system response (recovery), b_0_ is a constant that fixes the response at the central point of the experiment, bi is the fitting parameter for the linear effect term, and b_ii_, b_ij_ and b_ijk_ are the fitting parameters for the interaction effects terms. These fitting parameters allow the determination and comparison of the effects of each independent variable on recovery because of normalization. X_i_, X_j_ and X_k_ represent the normalized independent variables previously defined. Statistical calculations and analysis were performed using the Excel statistical module.

Tables S1 and S2 summarize the structure of the experimental design case by case.

### 3.5. Chromatographic Analysis

Chromatographic analysis was performed with an HPLC system (series 1100; Agilent Technologies, Palo Alto, USA) coupled with a DAD and a single-quadrupole mass spectrometer equipped with an ESI interface. The separation was achieved at 20 °C on a Phenomenex Kinetex^®^ 2.6 µm C18 100 Å (100 × 4.6 mm) column protected by an AJO-4287 guard cartridge. Elution solvents were ultrapure water acidified with 0.1% formic acid (A) and acetonitrile (B), and the elution was performed at a flow rate of 0.7 mL min^−1^ using the following gradient method: 50% B at 0 min, increase 50–95% B in 3 min and hold at 95% for 4 min.

MS quantification was performed using the most intense product ion (Figure S1), and the mass spectrometer operated in positive mode under the following conditions: nebulizer pressure: 35 psi; drying gas flow: 12.0 L min^−1^; drying gas temperature: 350 °C; gain: 1; nozzle voltage: 3000 V and fragmentor voltage: 150 V.

To evaluate the matrix effect in MS, the response of each of the six target compounds in the extraction solvent was compared with the response of these compounds in blank soil extracts within the linear concentration range (0.005–0.1 mg L^−1^). Blank soil extracts were obtained from the optimized extraction conditions with both QuEChERS extraction kits (i.e., citrate and acetate) alone and in combination with the clean-up steps tested (i.e., PSA and PSA-C18). The matrix effect on the target compounds was studied according to Equation 2 by comparing the slopes between the calibration curves in the extraction solvent and in the soil matrix.

(2) $$\mathrm{ME}(\%)=\left(\frac{S_{m}-S_{s}}{S_{s}}\right)\cdot 100$$

where ME is the matrix effect, S_m_ is the slope in the soil matrix and S_s_ is the slope in the extraction solvent. ME values below 0% indicate signal suppression, whereas values above 0% indicate signal enhancement. Notably, an ME > |20%| is commonly considered to have a significant impact on the performance of the analytical method.

## 4. Conclusions

A full 3^5^ second-order face-centered design of experiments was performed to examine in detail the effects of five independent variables, i.e., ET, WC, EV, SW and AM, on two different QuEChERS extraction systems, i.e., citrate and acetate, to efficiently recover three CHD herbicides and three of their byproducts from agricultural soil. This study involved 90 different experimental conditions between both systems, and the empirical mathematical models were found to fit well with the experimental results, yielding the following main results: highly polar and protic extraction media favor the target compound recoveries, while the AM exerts a significant and negative influence. Sethoxydim-oxazole follows the opposite rules. In general, the influence of the independent variables follows the order AM > SW > WC ≥ EV >> ET. A proper set of independent variables was selected, which enables recoveries close to 100% for the six target compounds studied: SW = 3.23 g, AM = 52.8%, EV = 8 mL, WC = 3.8 mL and ET = 1.8 min for citrate QuEChERS and SW = 3.96 g, AM = 57.0%, EV = 9.4 mL, WC = 4.0 mL and ET = 2.8 min for acetate QuEChERS.

QuEChERS does not readily achieve good recoveries with highly polar pesticides with log K_ow_ < −2 as a result of their poor or no partition into the organic phase. The aforementioned conclusions indicate that both QuEChERS versions, modified by introducing an additional solvent into the extraction system, i.e., methanol (polar/protic solvent), are good options for the simultaneous determination of pesticides with different behavior toward acetonitrile (polar/aprotic solvent) alone. In view of these promising results, an assessment of these QuEChERS versions over simultaneous residue recoveries of highly polar pesticides (e.g., fosetyl, ethephon and glyphosate) and nonpolar pesticides (e.g., pyrethroids and organophosphates such as chlorpyrifos) should be the next step toward the global use of these new QuEChERS approaches.

It is suggested that if nonsignificant terms want to be removed from the polynomial models, then a pertinent justification should be provided. In this particular case, the equation for acetate QuEChERS extraction can be simplified without great deviations relative to the experimental results. However, the results with the citrate QuEChERS suggest that all terms should be kept in the equation.

The extraction process was further studied by assessing the matrix effect observed when mass spectrometry instruments were used. Citrate QuEChERS in combination with the PSA + C18 clean-up step is more efficient in reducing the matrix effect than the acetate version with a clean-up step.

In summary, this methodology would allow optimizing the analytical procedure to analyze pesticide residues that possess different behavior during the extraction step. The application of chemometrics models can minimize analysis time, size of sample and solvent expense without compromising the analytic performance that will improve the accuracy of the method and thus the risk assessment of pesticides.

## Figures and Tables

**Table 1:** Polynomial model for citrate QuEChERS extraction of CHD residues from soil. Different letters indicate the factor significance at the ^a^ 95%, ^b^ 90%, ^c^ 80% and ^d^ 70% confidence levels.

Fitting Parameters	Alloxydim	Sethoxydim	Profoxydim	Deallyloxylated-alloxydim	Deethoxylated-sethoxydim	Sethoxydim-oxazole
b_0_	101.6 ^a^	106.9 ^a^	105.8 ^a^	100.3 ^a^	103.0 ^a^	103.7 ^a^
b_SW_	−0.8269	1.751	−2.371 ^a^	−0.5147	0.3115	−1.607 ^c^
b_AM_	−4.959 ^a^	−4.854 ^a^	−6.367 ^a^	−2.014 ^a^	−2.067 ^a^	10.12 ^a^
b_EV_	0.8366	1.933 ^c^	3.194 ^a^	0.9733 ^d^	1.417 ^c^	4.717 ^a^
b_WC_	−0.6412	−0.3913	−1.231 ^c^	0.5237	0.5356	1.105
b_ET_	0.9447 ^d^	0.3545	0.8743	1.373 ^c^	1.370 ^c^	0.3711
b_SW-AM_	−0.02532	0.2443	−0.3971	0.6546	0.5940	3.398 ^a^
b_SW-EV_	0.6864 ^d^	2.109 ^c^	−0.5446	1.497 ^c^	1.537 ^c^	2.265 ^b^
b_SW-WC_	−1.263 ^c^	0.006918	−0.6714	−0.9634	−0.7483	0.03950
b_SW-ET_	1.381 ^c^	0.8230	2.471 ^a^	1.489 ^c^	1.656 ^c^	0.8718
b_AM-EV_	−1.804 ^b^	−1.713 ^d^	−2.164 ^a^	−1.146 ^d^	−1.162 ^d^	−4.017 ^a^
b_AM-WC_	1.396 ^c^	1.685 ^d^	2.381 ^a^	1.710 ^b^	1.882 ^b^	0.8537
b_AM-ET_	−0.6283	−0.6910	−0.8073	−0.4672	−0.3677	0.3240
b_EV-WC_	0.06119	1.099	−0.4256	−0.6932	−0.7470	−0.9536
b_EV-ET_	−0.8625	−1.158	−0.7665	−1.217 ^c^	−1.651 ^c^	−1.030
b_WC-ET_	0.3770	0.7965	0.02497	0.6367	0.7979	1.111
b_SW-AM-EV_	−0.5365	−0.5793	−0.7477	−0.2786	0.001259	−0.5633
b_SW-AM-WC_	0.1645	−0.09995	1.413 ^c^	0.7606	1.248 ^d^	1.142
b_SW-AM-ET_	−0.6347	−0.6291	−0.6368	−0.5067	−0.4016 ^c^	−0.2854
b_SW-EV-WC_	0.6929	1.650 ^d^	0.2431	0.2161	0.1957	0.4587
b_SW-EV-ET_	−0.8404	−1.135	−0.04247	−1.062 ^d^	−1.513 ^d^	−0.6957
b_SW-WC-ET_	−0.2049	0.1874	−0.6880	−0.2849	−0.2937	0.1272
b_AM-EV-WC_	−1.505 ^c^	−2.514 ^b^	−2.980 ^a^	−1.443 ^c^	−2.404 ^a^	−2.534 ^a^
b_AM-EV-ET_	0.09265	−0.5385	0.03543	−0.09737	−0.5561	−0.6266
b_AM-WC-ET_	−0.6797	−0.3485	−0.5506	−0.6236	−0.6813	−0.2181
b_EV-WC-ET_	−0.3661	−0.3285	−0.1668	−0.5718	−0.7661	−0.3618
b_SW-SW_	−0.05830	−6.134 ^d^	5.027 ^c^	−0.6877	−1.293	−2.781
b_AM-AM_	−4.073 ^d^	−3.410	−5.206 ^c^	−2.465	−2.349	−10.91 ^a^
b_EV-EV_	−1.734	−2.347	−3.375	−1.115	−2.028	−1.976
b_WC-WC_	−2.133	−1.699	−3.023	−1.159	−1.306	−0.9245
b_ET-ET_	−0.1019	−0.2822	−0.8741	−0.9409	−0.5995	−1.470
R	0.9228	0.8905	0.9520	0.8761	0.8837	0.9674

**Table 2:** Polynomial model for acetate QuEChERS extraction of CHD residues from soil. Different letters indicate the factor significance at the ^a^ 95%, ^b^ 90%, ^c^ 80% and ^d^ 70% confidence levels.

Fitting Parameters	Alloxydim	Sethoxydim	Profoxydim	Deallyloxylated-alloxydim	Deethoxylated-sethoxydim	Sethoxydim-oxazole
b_0_	98.50 ^a^	96.30 ^a^	99.42 ^a^	96.52 ^a^	96.65 ^a^	93.59 ^a^
b_SW_	−2.046 ^a^	−1.623 ^a^	−0.9979 ^a^	−1.397 ^a^	−1.233 ^a^	−5.374 ^a^
b_AM_	−4.199 ^a^	−6.918 ^a^	−5.900 ^a^	−0.5917 ^c^	−0.6606 ^c^	14.45 ^a^
b_EV_	−0.3586	−0.4540 ^c^	−0.2489	−0.3171	−0.8767 ^b^	3.544 ^a^
b_WC_	0.7559 ^c^	0.4174	0.7932 ^b^	0.7751 ^b^	1.075 ^a^	2.006 ^a^
b_ET_	0.2162	0.3104	−0.04629	0.7781 ^b^	0.5730 ^d^	0.6799 ^c^
b_SW-AM_	−1.001 ^c^	0.006950	1.500 ^a^	0.4094	0.1525	2.818 ^a^
b_SW-EV_	−0.7845 ^c^	−1.148 ^a^	0.2673	−0.6982 ^c^	−0.6762 ^c^	−0.4862 ^d^
b_SW-WC_	1.452 ^a^	1.084 ^a^	1.852 ^a^	1.181 ^a^	0.9806 ^a^	1.086 ^a^
b_SW-ET_	0.1732	−0.08985	0.1410	−0.03353	−0.05905	−0.05778
b_AM-EV_	−0.5958	−0.7734 ^b^	−0.5355 ^c^	−1.098 ^a^	−1.141 ^a^	−5.220 ^a^
b_AM-WC_	−0.8033 ^c^	−0.7921 ^b^	0.3970	−0.5502	−0.4931	−1.827 ^a^
b_AM-ET_	−1.327 ^a^	−1.159 ^a^	−0.8263 ^a^	−0.9402 ^b^	−1.046 ^a^	−1.023 ^a^
b_EV-WC_	0.7362 ^d^	0.8608 ^c^	0.6544 ^c^	0.5351 ^d^	0.6066 ^c^	−0.08351
b_EV-ET_	0.3184	−0.1492	−0.2950	0.09183	−0.1445	−0.2852
b_WC-ET_	0.2434	−0.06107	−0.1582	0.07877	0.1107	0.7931 ^b^
b_SW-AM-EV_	−0.08380	−0.6281 ^c^	−1.232 ^a^	−0.2973	−0.6082 ^c^	−0.7774 ^b^
b_SW-AM-WC_	−0.4183	−0.8071 ^b^	−1.498 ^a^	−0.6732 ^c^	−0.8846 ^b^	−0.8068 ^b^
b_SW-AM-ET_	0.2012	−0.04719	−0.4896 ^d^	−0.08773	−0.1503	−0.5721 ^c^
b_SW-EV-WC_	−0.2389	0.1304 ^d^	0.6455 ^c^	0.07467 ^d^	0.02193 ^d^	0.4043
b_SW-EV-ET_	0.1344	0.5538	0.4635	0.8739 ^b^	0.5816	0.02332
b_SW-WC-ET_	0.2797	0.5672 ^d^	0.7497 ^b^	0.2862	0.5585 ^d^	−0.1483
b_AM-EV-WC_	0.05389	−0.2829	−0.4543 ^d^	−0.5252 ^d^	−0.5960 ^c^	0.2494
b_AM-EV-ET_	0.2714	0.04895	0.1469	−0.1186	−0.3965	−0.1905
b_AM-WC-ET_	0.2759	−0.1020	0.1953	0.09603	0.2365	−0.2606
b_EV-WC-ET_	−0.3355	−0.07306	0.1628	−0.1769	−0.08688	0.2886
b_SW-SW_	0.1189	−0.1245	1.538 ^d^	−1.291	−1.249	−0.7618
b_AM-AM_	−7.183 ^a^	−8.683 ^a^	−7.731 ^a^	−3.467 ^a^	−3.124 ^b^	−12.42 ^a^
b_EV-EV_	3.876 ^b^	3.331 ^a^	0.7742	2.272 ^c^	2.575 ^c^	1.251
b_WC-WC_	−1.693	−0.7284	−0.1338	−0.5212	−0.9923	−0.3232
b_ET-ET_	1.262	1.740 ^d^	1.222	2.566 ^c^	1.767 ^d^	1.881 ^d^
R	0.9458	0.9829	0.9830	0.9002	0.9085	0.9964

**Table 3:** Range of deviations between the experimental recoveries and the polynomial model results, together with the goodness of fit, at the minimum confidence level of the polynomial model regression parameters shown in Table 1 and Table 2.

Target Compound		Citrate QuEChERS–Confidence Levels					Acetate QuEChERS–Confidence Levels				
		95%	90%	80%	70%	0%	95%	90%	80%	70%	0%
Alloxydim	Deviation	25.5−0.0	23.7−0.0	18.2−0.3	15.5−0.0	6.5−0.1	6.3−0.1	6.1−0.0	5.8−0.0	5.1−0.0	4.4−0.0
	R	0.6156	0.6529	0.7336	0.8623	0.9228	0.8617	0.8820	0.9187	0.9254	0.9458
Sethoxydim	Deviation	26.2−0.1	26.2−0.1	22.1−0.2	15.9−0.0	13.0−0.1	5.4−0.0	5.0−0.0	4.3−0.0	3.1−0.0	2.8−0.0
	R	0.4977	0.4977	0.5606	0.8385	0.8905	0.9511	0.9696	0.9723	0.9796	0.9829
Profoxydim	Deviation	14.4−0.4	14.4−0.4	10.2−0.1	10.2−0.1	6.5−0.0	5.5−0.0	5.5−0.0	5.5−0.0	4.2−0.0	2.5−0.1
	R	0.8404	0.8404	0.9177	0.9177	0.9520	0.9487	0.9592	0.9663	0.9769	0.9830
Deallyloxylated-alloxydim	Deviation	24.8−0.1	23.1−0.0	16.1−0.0	11.9−0.0	6.8−0.1	6.7−0.1	4.9−0.1	3.7−0.0	3.7−0.0	3.0−0.0
	R	0.3113	0.4033	0.6230	0.6975	0.8761	0.5839	0.7345	0.8388	0.8730	0.9002
Deethoxylated-sethoxydim	Deviation	23.9−0.1	22.1−0.0	12.9−0.1	10.5−0.0	6.9−0.2	6.7−0.0	5.3−0.3	3.9−0.1	3.5−0.1	2.7−0.0
	R	0.4120	0.4775	0.6800	0.7143	0.8837	0.6288	0.7322	0.8390	0.8887	0.9085
Sethoxydim-oxazole	Deviation	14.4−0.0	12.2−0.1	11.4−0.0	11.4−0.0	11.1−0.1	4.3−0.1	4.3−0.0	4.3−0.0	4.0−0.0	4.2−0.0
	R	0.9280	0.9380	0.9434	0.9434	0.9674	0.9906	0.9933	0.9945	0.9955	0.9964

**Table 4:** Optimized conditions selected for the extraction of CHD residues from soil by citrate and acetate QuEChERS, together with the predicted and experimental results for the target compounds.

QuEChERS	Optimized Conditions ^a^	Recovery (%) ^b^											
		Alloxydim		Sethoxydim		Profoxydim		Deallyloxylated-alloxydim		Deethoxylated-sethoxydim		Sethoxydim-oxazole	
		Pred.	Exp.	Pred.	Exp.	Pred.	Exp.	Pred.	Exp.	Pred.	Exp.	Pred.	Exp.
Citrate	SW = 3.23 g AM = 52.8% EV = 8 mL WC = 3.8 mL ET = 1.8 min	98.9	100.5	100.0	98.8	100.1	97.1	99.3	102.7	100.0	99.4	99.7	97.5
Acetate	SW = 3.96 g AM = 57.0% EV = 9.4 mL WC = 4.0 mL ET = 2.8 min	99.9	101.2	97.6	101.9	99.8	100.5	99.9	102.1	99.9	100.3	97.7	96.5

^a^ SW: soil weight; AM: solvent extraction composition (acetonitrile/methanol); EV: solvent extraction volume; ET: extraction time; WC: water content. ^b^ Pred.: predicted values using the full equations of Table 1 and Table 2; Exp.: experimental values (*n* = 3).

## Supplementary Materials

Figure S1 and Tables S1–S4 were provided as supplementary material and are available online.

## References

[1] Tong Z., Wu Y.-C., Liu Q.-Q., Shi Y.-H., Zhou L.-J., Liu Z.-Y., Yu L.-S., Cao H.-Q. (2016). Multi-residue analysis of pesticide residues in crude pollens by UPLC-MS/MS. Molecules.

[2] Ccanccapa A., Masiá A., Navarro-Ortega A., Picó Y., Barceló D. (2016). Pesticides in the Ebro river basin: Occurrence and risk assessment. Environ. Pollut..

[3] Villaverde J.J., Sevilla-Morán B., López-Goti C., Alonso-Prados J.L., Sandín-España P. (2016). Trends in analysis of pesticide residues to fulfil the European Regulation (EC) No. 1107/2009. TrAC Trends Anal. Chem..

[4] Hvězdová M., Kosubová P., Košíková M., Scherr K.E., Šimek Z., Brodský L., Šudoma M., Škulcová L., Sáňka M., Svobodová M. (2018). Currently and recently used pesticides in Central European arable soils. Sci. Tot. Environ..

[5] Mantzos N., Karakitsou A., Hela D., Patakioutas G., Leneti E., Konstantinou I. (2014). Persistence of oxyfluorfen in soil, runoff water, sediment and plants of a sunflower cultivation. Sci. Tot. Environ..

[6] Bargańska Ż., Ślebioda M., Namieśnik J. (2014). Determination of pesticide residues in honeybees using modified QUEChERS sample work-up and liquid chromatography-tandem mass spectrometry. Molecules.

[7] Villaverde J.J., Sevilla-Morán B., López-Goti C., Alonso-Prados J.L., Sandín-España P. (2017). Computational methodologies for the risk assessment of pesticides in the European Union. J. Agric. Food Chem..

[8] Villaverde J.J., Sevilla-Morán B., López-Goti C., Calvo L., Alonso-Prados J.L., Sandín-España P. (2018). Photolysis of clethodim herbicide and a formulation in aquatic environments: Fate and ecotoxicity assessment of photoproducts by QSAR models. Sci. Tot. Environ..

[9] Sevilla-Morán B., Calvo L., López-Goti C., Alonso-Prados J.L., Sandín-España P. (2017). Photodegradation behaviour of sethoxydim and its comercial formulation Poast^®^ under environmentally-relevant conditions in aqueous media. Study of photoproducts and their toxicity. Chemosphere.

[10] Roberts T.R. (1998). Metabolic Pathways of Agrochemicals.

[11] Villaverde J.J., Santín-Montanyá I., Sevilla-Morán B., Alonso-Prados J., Sandín-España P. (2018). Assessing the effects of alloxydim phototransformation products by QSAR models and a phytotoxicity study. Molecules.

[12] You X., Liang L., Liu F. (2014). Dissipation and residues of clethodim and its oxidation metabolites in a rape-field ecosystem using QuEChERS and liquid chromatography/tandem mass spectrometry. Food Chem..

[13] Marek L.J., Koskinen W.C., Bresnahan G.A. (2000). LC/MS analysis of cyclohexanedione oxime herbicides in water. J. Agric. Food Chem..

[14] Sandín-España P., González-Blázquez J.J., Magrans J.O., García-Baudín J.M. (2002). Determination of herbicide tepraloxydim and main metabolites in drinking water by solid-phase extraction and liquid chromatography with UV detection. Chromatographia.

[15] Tsochatzis E.D., Tzimou-Tsitouridou R., Menkissoglu-Spiroudi U., Karpouzas D.G., Maria Papageorgiou G. (2012). Development and validation of an HPLC-DAD method for the simultaneous determination of most common rice pesticides in paddy water systems. Intern. J. Environ. Anal. Chem..

[16] Saha M., Harrison B., Collins L. GC-MSD method for the determination of tepraloxydim and its major metabolites residues in soil using conversion to the common analyte approach. Proceedings of the 220th ACS National Meeting.

[17] Anastassiades M., Lehotay S.J., Stajnbaher D., Schenck F.J. (2003). Fast and easy multiresidue method employing acetonitrile extraction/partitioning and dispersive soildphase extraction for the determination of pesticide residues in produce. J. AOAC Int..

[18] Romero-González R., Arrebola Liébanas F.J., López-Ruiz R., Garrido Frenich A. (2016). Sample treatment in pesticide residue determination in food by high-resolution mass spectrometry: Are generic extraction methods the end of the road?. J. AOAC Int..

[19] Łozowicka B., Rutkowska E., Jankowska M. (2017). Influence of QuEChERS modifications on recovery and matrix effect during the multi-residue pesticide analysis in soil by GC/MS/MS and GC/ECD/NPD. Environ. Sci. Pollut. Res..

[20] Berlioz-Barbier A., Vauchez A., Wiest L., Baudot R., Vulliet E., Cren-Olivé C. (2014). Multi-residue analysis of emerging pollutants in sediment using QuEChERS-based extraction followed by LC-MS/MS analysis. Anal. Bioanal. Chem..

[21] Azevedo de Brito W., Gomes Dantas M., Andrade Nogueira F.H., Ferreira da Silva-Júnior E., de Araújo-Júnior J.X., Mendonça de Aquino T., Nogueira Ribeiro Ê.A., da Silva Solon L., Soares Aragão C.F., Barreto Gomes A.P. (2017). Development and validation of HPLC-DAD and UHPLC-DAD methods for the simultaneous determination of guanylhydrazone derivatives employing a factorial design. Molecules.

[22] Santos S.A.O., Villaverde J.J., Silva C.M., Neto C.P., Silvestre A.J.D. (2012). Supercritical fluid extraction of phenolic compounds from *Eucalyptus globulus* Labill bark. J. Supercrit. Fluids.

[23] Villaverde J.J., Ligero P., Vega A.d. (2009). Bleaching *Miscanthus x giganteus* acetosolv pulps with hydrogen peroxide/acetic acid. Part 1: Behaviour in aqueous alkaline media. Bioresour. Technol..

[24] Bruzzoniti M.C., Checchini L., De Carlo R.M., Orlandini S., Rivoira L., Del Bubba M. (2014). QuEChERS sample preparation for the determination of pesticides and other organic residues in environmental matrices: A critical review. Anal. Bioanal. Chem..

[25] Lehotay S.J., Son K.A., Kwon H., Koesukwiwat U., Fu W., Mastovska K., Hoh E., Leepipatpiboon N. (2010). Comparison of QuEChERS sample preparation methods for the analysis of pesticide residues in fruits and vegetables. J. Chromatogr. A.

[26] Rashid A., Nawaz S., Barker H., Ahmad I., Ashraf M. (2010). Development of a simple extraction and clean-up procedure for determination of organochlorine pesticides in soil using gas chromatography-tandem mass spectrometry. J. Chromatogr. A.

[27] Correia-Sá L., Fernandes V.C., Carvalho M., Calhau C., Domingues V.F., Delerue-Matos C. (2012). Optimization of QuEChERS method for the analysis of organochlorine pesticides in soils with diverse organic matter. J. Sep. Sci..

[28] Fernandes V.C., Domingues V.F., Mateus N., Delerue-Matos C. (2013). Multiresidue pesticides analysis in soils using modified QuEChERS with disposable pipette extraction and dispersive solid-phase extraction. J. Sep. Sci..

[29] Ndongo G.A., Boyomo M.O., Owono P.A. (2018). Ab initio study of polar and non-polar aprotic solvents effects on some 3-hydroxychromones and 3-hydroxyquinolones derivatives. J. Mol. Model..

[30] Alam M.S., Ashokkumar B., Mohammed Siddiq A. (2018). The density, dynamic viscosity and kinematic viscosity of protic polar solvents (pure and mixed systems) studies: A theoretical insight of thermophysical properties. J. Mol Liq..

[31] De Carlo R.M., Rivoira L., Ciofi L., Ancillotti C., Checchini L., Del Bubba M., Bruzzoniti M.C. (2015). Evaluation of different QuEChERS procedures for the recovery of selected drugs and herbicides from soil using LC coupled with UV and pulsed amperometry for their detection. Anal. Bioanal. Chem..

[32] Paíga P., Delerue-Matos C. (2016). A throughput method using the quick easy cheap effective rugged safe method for the quantification of ibuprofen and its main metabolites in soils. J. Sep. Sci..

[33] Myers R.H., Montgomery D.C., Anderson-Cook C.M. (2016). Response Surface Methodology: Process and Product Optimization Using Designed Experiments.

[34] Vilela C., Santos S.A.O., Villaverde J.J., Oliveira L., Nunes A., Cordeiro N., Freire C.S.R., Silvestre A.J.D. (2014). Lipophilic phytochemicals from banana fruits of several *Musa* species. Food Chem..

[35] Mantzos N., Karakitsou A., Zioris I., Leneti E., Konstantinou I. (2013). QuEChERS and solid phase extraction methods for the determination of energy crop pesticides in soil, plant and runoff water matrices. Int. J. Environ. Anal. Chem..

[36] Mei M., Du Z.-X., Cen Y. (2011). QuEChERS-Ultra-performance liquid chromatography tandem mass spectrometry for determination of five currently used herbicides. Chin. J. Anal. Chem..

[37] Kwon H., Lehotay S.J., Geis-Asteggiante L. (2012). Variability of matrix effects in liquid and gas chromatography-mass spectrometry analysis of pesticide residues after QuEChERS sample preparation of different food crops. J. Chromatogr. A.

[38] Kmellár B., Fodor P., Pareja L., Ferrer C., Martínez-Uroz M.A., Valverde A., Fernández-Alba A.R. (2008). Validation and uncertainty study of a comprehensive list of 160 pesticide residues in multi-class vegetables by liquid chromatography-tandem mass spectrometry. J. Chromatogr. A.

[39] Sandín-España P., Sevilla-Morán B., Calvo L., Mateo-Miranda M., Alonso-Prados J.L. (2013). Photochemical behavior of alloxydim herbicide in environmental waters. Structural elucidation and toxicity of degradation products. Microchem. J..

[40] Sheldrick B.H., Wang C., Carter M.R. (1993). Particle size distribution. Soil Sampling and Methods of Analysis.

[41] Nelson D.W., Sommers L.E., Sparks D.L., Page A.L., Helmke P.A., Loeppert R.H., Soltanpour P.N., Tabatabai M.A., Johnston C.T., Sumner M.E. (1996). Total carbon, organic carbon, and organic matter. Methods of Soil Analysis, Part 3: Chemical Methods.

[42] Bremner J.M., Mulvaney C.S., Page A.L., Miller R.H., Keeney D.R. (1982). Nitrogen-total. Methods of Soil Analysis, Part 2: Chemical and Microbiological Properties.

