# Supplementary material for: Computational-Based Study of QuEChERS Extraction of Cyclohexanedione Herbicide Residues in Soil by Chemometric Modeling
Source: Molecules. 2018 Aug 11;23(8):2009. doi: 10.3390/molecules23082009 (PMC6222645; doi:10.3390/molecules23082009)
Supplement: Supplementary file 1 [file molecules-23-02009-s001.zip › molecules-340007-supplementary.pdf]

Computational-Based Study of QuEChERS Extraction of Cyclohexanedione Herbicide Residues in Soil by Chemometric Modeling

Juan José Villaverde, Beatriz Sevilla-Morán, Carmen López-Goti, José Luis Alonso-Prados and Pilar Sandín-España *

Unit Plant Protection Products, DTEVPF, INIA. Crta. La Coruña, Km.7.5, 28040 Madrid, Spain; juanjose.villaverde@inia.es (J.J.V.); bsmoran@inia.es (B.S.-M.); lgoti@inia.es (C.L.G.); prados@inia.es (J.L. A.-P.)

* Correspondence: sandin@inia.es; Tel.: +34-91-347-8709

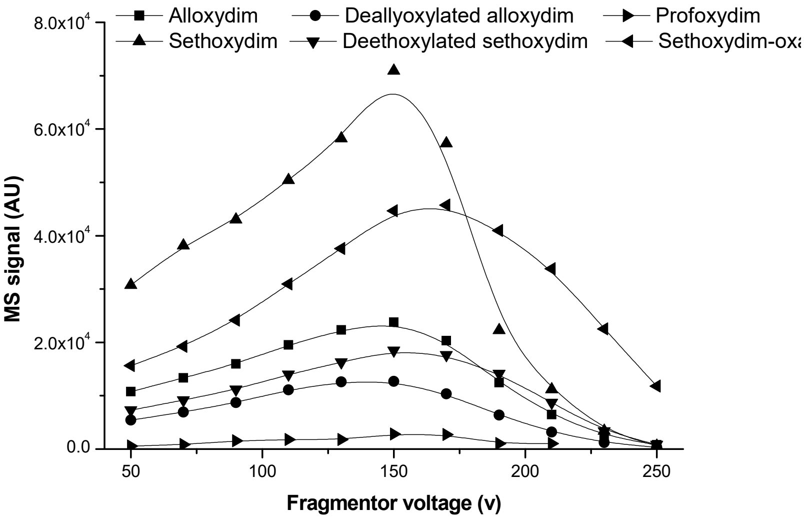

Figure S1. Intensities of the product ions selected versus fragmentor voltages in the mass spectrometer operating under the conditions described in Section 2.5.

Table S1. Experimental design structure and results for the citrate QuEChERS (SW: soil weight; AM: solvent extraction composition (acetonitrile/methanol); EV: solvent extraction volume; ET: extraction time; WC: water content).

Nº exp.	SW (g)	AM (%)	EV (mL)	WC (mL)	ET (min)	Deallyloxylated Alloxydim	Deethoxylated Sethoxydim	Sethoxydim-Oxazole	Alloxydim	Sethoxydim	Profoxydim
1	2.4	5	8	1.5	1	96.5	98.1	75.3	99.4	98.9	106.1
2	2.4	5	8	1.5	3	95.5	95.4	72.4	98.6	98.6	103.5
3	2.4	5	8	4.5	1	99.2	98.5	78.4	99.7	95.6	101.1
4	2.4	5	8	4.5	3	96.3	94.9	71.3	94.0	89.0	94.8
5	2.4	5	16	1.5	1	96.5	96.0	84.5	100.4	97.4	110.7
6	2.4	5	16	1.5	3	98.2	99.1	83.8	101.1	99.0	112.0
7	2.4	5	16	4.5	1	97.4	100.3	88.3	99.7	95.0	114.1
8	2.4	5	16	4.5	3	98.1	100.3	89.2	101.4	96.9	110.0
9	2.4	95	8	1.5	1	91.0	91.6	91.4	88.7	86.0	90.8
10	2.4	95	8	1.5	3	87.6	86.7	89.3	85.4	82.5	86.5
11	2.4	95	8	4.5	1	93.4	93.5	94.5	91.3	88.0	93.0
12	2.4	95	8	4.5	3	100.8	102.9	104.3	97.3	94.7	98.8
13	2.4	95	16	1.5	1	92.4	95.4	99.6	90.9	88.9	106.4
14	2.4	95	16	1.5	3	86.6	88.4	92.5	85.6	82.3	93.1
15	2.4	95	16	4.5	1	90.9	90.6	90.5	89.1	84.5	95.5
16	2.4	95	16	4.5	3	91.9	90.6	89.8	87.8	83.6	92.7
17	5.8	5	8	1.5	1	95.1	98.3	63.7	99.1	102.5	103.7
18	5.8	5	8	1.5	3	95.9	99.1	59.3	100.1	98.9	108.7
19	5.8	5	8	4.5	1	72.7	73.0	46.9	74.9	72.6	76.6
20	5.8	5	8	4.5	3	99.3	100.7	63.9	100.1	96.3	100.0
21	5.8	5	16	1.5	1	98.2	101.6	82.0	100.7	100.0	108.4
22	5.8	5	16	1.5	3	101.1	102.0	78.8	103.3	101.4	113.2
23	5.8	5	16	4.5	1	97.3	99.9	83.6	100.7	109.9	103.6
24	5.8	5	16	4.5	3	97.6	101.0	82.9	100.0	108.3	108.2
25	5.8	95	8	1.5	1	78.8	76.4	84.5	82.0	81.1	77.5
26	5.8	95	8	1.5	3	93.3	94.5	92.3	93.1	91.5	90.6
27	5.8	95	8	4.5	1	96.1	97.5	100.0	92.0	91.5	99.4
28	5.8	95	8	4.5	3	95.2	100.1	102.4	86.4	88.6	90.7
29	5.8	95	16	1.5	1	92.9	96.4	100.5	87.1	94.2	86.3
30	5.8	95	16	1.5	3	94.6	96.9	99.3	89.0	87.5	95.1

31	5.8	95	16	4.5	1	92.4	96.9	99.6	84.9	92.9	86.5
32	5.8	95	16	4.5	3	92.0	93.3	99.9	85.5	90.8	88.1
33	4.1	50	12	3	2	98.5	97.9	94.9	100.6	98.2	104.9
34	4.1	50	12	3	2	99.0	100.6	96.4	100.6	97.9	104.9
35	4.1	50	12	3	2	95.0	96.0	92.6	97.0	93.8	102.1
36	2.4	50	12	3	2	99.6	100.7	104.0	100.7	98.5	115.5
37	5.8	50	12	3	2	101.9	106.3	104.7	104.0	110.7	107.5
38	4.1	5	12	3	2	100.8	104.3	87.4	103.9	114.0	107.7
39	4.1	95	12	3	2	97.1	100.6	105.1	92.7	100.6	94.9
40	4.1	50	8	3	2	99.5	102.6	102.6	99.1	103.6	100.0
41	4.1	50	16	3	2	101.1	103.0	107.7	102.2	113.1	106.2
42	4.1	50	12	1.5	2	99.6	103.5	105.6	101.4	109.4	104.8
43	4.1	50	12	4.5	2	100.9	103.5	106.8	99.1	108.6	102.1
44	4.1	50	12	3	1	98.7	101.8	103.3	100.3	109.9	104.0
45	4.1	50	12	3	3	102.2	106.6	108.0	104.3	111.0	107.2

Table S2. Experimental design structure and results for the acetate QuEChERS (SW: soil weight; AM: solvent extraction composition (acetonitrile/methanol); EV: solvent extraction volume; ET: extraction time; WC: water content).

Nº exp.	SW (g)	AM (%)	EV (mL)	WC (mL)	ET (min)	Deallyloxylated Alloxydim	Deethoxylated Sethoxydim	Sethoxydim-Oxazole	Alloxydim	Sethoxydim	Profoxydim
1	2.4	5	8	1.5	1	95.6	95.4	65.0	96.3	97.3	103.5
2	2.4	5	8	1.5	3	103.5	103.6	67.0	105.1	106.5	112.9
3	2.4	5	8	4.5	1	93.1	94.3	68.6	96.7	95.8	102.1
4	2.4	5	8	4.5	3	96.9	96.1	73.4	98.7	98.5	99.9
5	2.4	5	16	1.5	1	97.3	94.6	85.0	98.9	99.4	104.8
6	2.4	5	16	1.5	3	99.5	98.3	82.8	101.7	101.1	104.4
7	2.4	5	16	4.5	1	100.3	99.5	84.0	101.9	102.3	102.1
8	2.4	5	16	4.5	3	100.9	99.1	90.1	101.9	101.7	97.3
9	2.4	95	8	1.5	1	97.1	96.7	100.6	101.6	87.9	88.2
10	2.4	95	8	1.5	3	96.4	95.2	100.3	90.4	85.7	86.6
11	2.4	95	8	4.5	1	96.1	96.4	100.0	92.0	85.7	90.1
12	2.4	95	8	4.5	3	99.1	99.8	103.0	92.3	85.7	89.9
13	2.4	95	16	1.5	1	97.3	96.0	100.7	93.6	87.9	89.2
14	2.4	95	16	1.5	3	97.3	95.2	100.7	94.9	88.4	88.4
15	2.4	95	16	4.5	1	95.8	96.4	99.3	94.3	87.9	89.6
16	2.4	95	16	4.5	3	94.4	94.1	100.0	93.0	84.9	88.8
17	5.8	5	8	1.5	1	92.7	92.7	43.6	95.8	95.7	95.4
18	5.8	5	8	1.5	3	90.4	90.8	46.5	94.0	93.7	93.8
19	5.8	5	8	4.5	1	95.1	95.3	54.2	99.3	97.3	96.1
20	5.8	5	8	4.5	3	98.8	99.2	60.0	103.2	101.5	101.1
21	5.8	5	16	1.5	1	87.7	88.3	61.8	91.7	91.2	93.6
22	5.8	5	16	1.5	3	95.0	93.3	65.4	95.5	94.6	96.0
23	5.8	5	16	4.5	1	95.2	94.9	71.6	97.2	97.0	102.4
24	5.8	5	16	4.5	3	101.7	102.6	78.1	104.4	103.6	108.5
25	5.8	95	8	1.5	1	97.2	97.0	98.1	89.3	88.1	91.5
26	5.8	95	8	1.5	3	96.9	97.5	98.3	89.0	87.6	90.6
27	5.8	95	8	4.5	1	97.8	97.6	98.1	90.2	86.0	93.3
28	5.8	95	8	4.5	3	95.9	96.8	97.2	88.7	83.7	90.9
29	5.8	95	16	1.5	1	90.8	91.1	93.6	84.9	80.3	87.3
30	5.8	95	16	1.5	3	90.2	87.3	87.8	83.0	78.0	84.0

31	5.8	95	16	4.5	1	92.7	91.9	93.6	88.1	82.3	91.7
32	5.8	95	16	4.5	3	94.3	91.6	93.3	86.7	80.5	89.2
33	4.1	50	12	3	2	93.5	94.3	92.3	96.4	94.6	99.6
34	4.1	50	12	3	2	94.8	95.1	92.3	96.1	94.9	99.3
35	4.1	50	12	3	2	94.8	94.5	91.9	96.1	95.1	100.7
36	2.4	50	12	3	2	95.8	95.7	95.0	98.0	96.5	101.5
37	5.8	50	12	3	2	96.3	96.6	91.7	101.0	96.9	100.0
38	4.1	5	12	3	2	96.7	96.8	69.7	98.5	97.3	99.6
39	4.1	95	12	3	2	91.0	91.8	93.7	85.8	79.0	83.5
40	4.1	50	8	3	2	98.7	99.6	92.1	104.0	100.1	99.9
41	4.1	50	16	3	2	100.5	100.3	98.6	102.5	100.2	100.1
42	4.1	50	12	1.5	2	95.2	94.4	93.4	96.3	94.6	97.0
43	4.1	50	12	4.5	2	98.4	98.4	94.2	99.1	97.7	101.2
44	4.1	50	12	3	1	101.2	100.5	97.5	102.4	100.1	101.8
45	4.1	50	12	3	3	98.6	97.8	94.5	98.9	97.1	99.1

Table S3. Chromatographic resolution of deallyloxylated alloxydim (A), deethoxylated sethoxydim (B), sethoxydim-oxazole (C), alloxydim (D), sethoxydim (E) and profoxydim (F) using three different chromatographic columns (i.e., Kinetex, Nova-Pak and Atlantis T3).

**Chromatographic Column**	**Chromatographic Resolution**					**Run Time (min)**
	A-B	B-C	C-D	D-E	E-F	
Kinetex	11.9	7.0	7.0	9.6	7.0	7.0
Nova-Pak	10.6	7.5	5.5	8.4	7.7	7.5
Atlantis T3	8.9	11.0	5.3	12.1	8.6	11.0

Table S4. HPLC-DAD-MS features of the target compounds.

Target Compound	t_R (min)	λ_{max} (nm)	Measured Mass m/z (Relative Abundance)	Major Product Ions
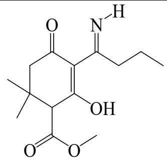<br>Deallyloxylated alloxydim	2.517	290 (max)*<br>252	290 (38)<br>268 (100)*<br>236 (43)<br>208 (18)<br>180 (5)	$[M+Na]^+$<br>$[M H]^+$<br>$[M+H]^+ – [CH_3OH]$<br>$[M+H]^+ – [CH_3OH] – [CO]$<br>$[M+H]^+ – [CH_3OH] – [CO] – [CO]$
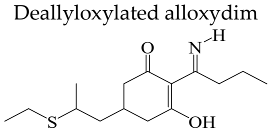<br>Deethoxylated sethoxydim	3.639	290 (max)*<br>248	306 (3)<br>284 (100)*<br>266 (12)<br>222 (9)	$[M+Na]^+$<br>$[M+H]^+$<br>$[M+H]^+ – [H_2O]$<br>$[M+H]^+ – [CH_3-CH_2-SH]$
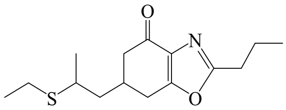<br>Sethoxydim-oxazole	4.466	248*<br>210 (max)	304 (13)<br>282 (100)*<br>220 (3)<br>178 (2)	$[M+Na]^+$<br>$[M+H]^+$<br>$[M+H]^+ – [CH_3-CH_2-SH]$<br>$[M+H]^+ – [CH_3-CH_2-S-CH-(CH_3)_2]$
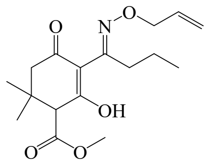<br>Alloxydim	5.121	290 (shoulder)<br>258 (max)*<br>214	346 (17)<br>324 (100)*<br>266 (19)<br>234 (6)<br>206 (3)	$[M+Na]^+$<br>$[M+H]^+$<br>$[M+H]^+ – [HO-CH_2-CH=CH_2]$<br>$[M+H]^+ – [HO-CH_2-CH=CH_2] – [CH_3OH]$<br>$[M+H]^+ – [HO-CH_2-CH=CH_2] – [CH_3OH – CO]$
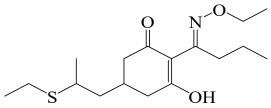<br>Sethoxydim	5.983	286 (shoulder)<br>252 (max)*<br>210	350 (2)<br>328 (100)*<br>282 (17)<br>220 (3)<br>178 (4)	$[M+Na]^+$<br>$[M+H]^+$<br>$[M+H]^+ – [CH_3CH_2OH]$<br>$[M+H]^+ – [CH_3CH_2OH] – [CH_3CH_2SH]$<br>$[M+H]^+ – [CH_3CH_2OH] – [CH_3CH_2SCH(CH_3)_2]$
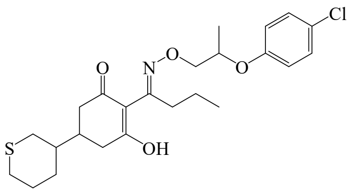<br>Profoxydim	6.617	282 (shoulder)<br>250 (shoulder)<br>230*<br>194 (max)	488 (25)<br>466 (100)*<br>280 (5)	$[M+Na]^+$<br>$[M+H]^+$<br>$[M+H]^+ – [C_6H_5(Cl)OCH(CH_3)CH_2OH]$

*DAD and MSD signals selected for quantification purposes.
